# Maternal COVID-19 exposure and placental characteristics

**DOI:** 10.1371/journal.pone.0302682

**Published:** 2024-05-23

**Authors:** Ghassan Allo, Alexandra R. Sitarik, Ashley Redding, Chad M. Coleman, Andrea E. Cassidy-Bushrow, Arthur Gaba, Jennifer K. Straughen

**Affiliations:** 1 Department of Pathology, Henry Ford Hospital, Detroit, MI, United States of America; 2 Department of Obstetrics, Gynecology and Reproductive Biology, College of Human Medicine, Michigan State University, East Lansing, MI, United States of America; 3 Department of Public Health Sciences, Henry Ford Health, Detroit, MI, United States of America; Universita Politecnica delle Marche, ITALY

## Abstract

**Introduction:**

The impact of COVID-19 on the placenta is poorly described, particularly among minority women.

**Materials and methods:**

This is a retrospective case-control study. Micro- and macroscopic placental pathologic findings were compared for 15 COVID-19 positive and 36 negative mothers. Cases and controls were frequency matched on gestational age, race, maternal comorbidities, and delivery type. Data from the electronic medical record were supplemented with independent review of microscopic slides.

**Results:**

Placentas from cases and controls were similar except the median distance from the site of the cord insertion to the nearest disk margin was statistically significantly shorter among placentas from COVID-19 positive cases (3.5 versus 6.0 cm, p = 0.006). Case status was not associated with an increased risk of placental pathologies.

**Conclusion:**

There are few pathologic differences between placentas of COVID-19 positive and negative mothers. Additional studies are needed to investigate the role of timing of infection.

## Introduction

COVID-19 is a respiratory illness caused by infection with SARS-CoV-2 [1]. This single-stranded RNA virus induces its pathogenic effect through its direct effect on infected tissue and through immune-mediated mechanism that may result in a “cytokine storm” in more advanced disease stages [2]. The virus also infects endothelial cells inducing neutrophilic and monocytic infiltration and thromboembolic events [2]. Given the relationship between placental inflammation and both immediate and long-term pregnancy and health outcomes, such as preterm birth and offspring neurodevelopment, it is essential that the impact of maternal COVID-19 illness on the placenta be more fully characterized.

At present, few studies have examined placental involvement by SARS-CoV-2, though there is a growing interest in transplacental vertical transmission. Some authors have reported evidence of viral RNA or proteins within placental tissue and swabs from newborns, albeit rare [3–14]. However, others failed to replicate these findings [15–19]. Further, one study suggests that SARS-CoV-2 is unlikely to pass to through the placenta *in utero* [20]. Despite the controversy surrounding vertical transmission, it is unknown whether the virus impacts placental function or histopathology. Changes in placental function or histopathology regardless of actual infection may affect pregnancy outcomes or long-term offspring health as suggested by the Developmental Origins of Health and Disease Hypothesis. Histopathologic findings in the placenta have been found to be associated with adverse pregnancy outcomes such as preterm birth [21] and offspring neurodevelopmental outcomes [22], thus it is imperative that placental histopathology associated with COVID-19 infection in pregnant women be characterized. At present, placental pathologic findings from COVID-19 positive mothers are poorly described and studies including minority women or reporting patient race are decidedly rare, despite the literature suggesting that rates of COVID-19 infection were higher for racial and ethnic minority groups [23,24] and COVID-19-related mortality [25]. Among the largest studies to date, Baergen and Heller reported on 20 cases with COVID-19 and noted a high prevalence of both fetal vascular malperfusion and fetal vascular thrombosis, yet with no control group, the significance is unknown [26]. Shanes et al. described placental pathology in 16 cases compared to > 17,000 historical controls and also noted elevated rates of maternal vascular malperfusion features and intervillous thrombi [27]. Other studies have also noted higher rates of maternal vascular malperfusion [15,28].

In this study, we report macroscopic and microscopic pathology of placentas from women tested positive for COVID-19 during pregnancy in comparison with those from pregnant women who tested negative from an urban diverse community.

## Materials and methods

This case-control study was approved by the Henry Ford Health Institutional Review Board (#13765). The need for consent was waived by the ethics committee. Cases and controls were identified programmatically through queries of the electronic medical record which were accessed between April 3, 2020 and June 30, 2020. All women who delivered a singleton infant between March 17, 2020 and June 10, 2020, at a Henry Ford Health hospital and were tested for COVID-19 near the time of delivery were eligible for inclusion in the study. COVID-19 testing of women was performed using in-house developed and validated reverse transcriptase polymerase chain reaction following US Food and Drug Administration regulations [29,30]. Some women had multiple tests. If there were multiple tests and one test was prior to delivery and one was after delivery, only the test prior to delivery was retained and used for determination of COVID-19 exposure status. Additionally, COVID-19 test results reported after delivery were only retained if they were within 5 days postpartum, as this likely reflects infection during pregnancy. Cases were defined as pregnant women with at least 1 positive COVID-19 test prior to delivery or within 5 days postpartum. Controls were defined as pregnant women who delivered at a study hospital during the study period who had negative COVID-19 test results prior to delivery or within 5 days postpartum. Dates of testing ranged from a gestational age of 31 weeks to 5 days postpartum.

During the study period, 25 women tested positive for COVID-19, delivered at a study hospital, and had their placenta sent to pathology for clinical indications. These 25 women were retained as cases. Consecutive deliveries during the study period where the placenta was sent to pathology for clinical indications and had a negative COVID-19 test were then selected as controls. Cases and controls were frequency matched on gestational age, race, presence of a maternal comorbidity, and delivery type. In total, 40 controls were identified, but 3 of the 40 controls were subsequently excluded as the COVID-19 testing occurred more than 5 days postpartum. To minimize confounding due to gestational age-related changes in the placenta, only placenta from viable births were included; one 16-week placenta was excluded for a final sample size of 25 cases and 36 controls. Clinical data and symptomatology at the time of testing were retrieved from the electronic medical records, and disease severity was classified according to previously established criteria [31]. All cases were assigned a study-specific identifier and the protected health information was redacted such that authors did not have access to information that could identify individual participants during or after data collection.

All placentas were handled according to standard clinical methods. After delivery, placentas were fixed in formalin, paraffin embedded, and representative sections were stained with hematoxylin and eosin. Macroscopic (gross) findings of all placentas were abstracted from the clinical pathology report in the electronic medical record. Microscopy was evaluated using Amsterdam criteria [32] by 2 expert gynecologic pathologists (GA and AG) blinded to COVID-19 status. Each of the two pathologists initially reviewed all cases independently. All microscopic discrepancies were discussed and resolved by the 2 pathologists and a single diagnosis was assigned.

Statistical analysis for the comparison of placentas from COVID-19 positive women to those who were COVID-19 negative was performed using R version 4.0.0 (R Foundation, Vienna, Austria). The Kruskal-Wallis test was used for continuous variables, while the chi-square test or Fisher’s exact test was used for categorical variables (when the expected frequencies were less than 5 in some cells). Statistical significance was set at 0.05.

## Results

The sociodemographic and clinical characteristics of cases and controls are summarized in Table 1. The entire study cohort was composed of 61 women, 18 to 48 years of age, who gave birth between 31 and 41 weeks of gestation (median 39 weeks). There was no difference between the case and control groups with regards to demographic or clinical characteristics including maternal age, race, maternal comorbidities, delivery type, gestational age, and newborn birth weight. A greater proportion of COVID-19 positive infants were female (68% versus 32%, p = 0.048). Among women who tested positive for COVID-19, 12 of 25 (48%) were symptomatic at the time of the test, having mild, moderate, severe, and critical disease in 4 (16%), 3 (12%), 1 (4%), and 4 (16%), respectively.

**Table 1:** Maternal and infant sociodemographic and clinical characteristics among COVID-19 positive and negative pregnant women.

	Total N = 61	Negative N = 36	Positive N = 25	p-value
	N (%) or Median (IQR) [Min, Max]			
Maternal age (years)	29 (8) [18, 48]	29 (6) [18, 40]	28 (9) [18, 48]	0.653
Maternal race				0.256
Black	23 (37.7%)	10 (27.8%)	13 (52.0%)	
White	19 (31.1%)	12 (33.3%)	7 (28.0%)	
Other	15 (24.6%)	11 (30.6%)	4 (16.0%)	
Unknown	4 (6.56%)	3 (8.33%)	1 (4.00%)	
Delivery type				0.657
C-section	26 (42.6%)	14 (38.9%)	12 (48.0%)	
Vaginal	35 (57.4%)	22 (61.1%)	13 (52.0%)	
Infant sex				0.048
Female	31 (50.8%)	14 (38.9%)	17 (68.0%)	
Male	30 (49.2%)	22 (61.1%)	8 (32.0%)	
Gestational age at COVID-19 test order (weeks)	38 (3) [31, 41]	39 (2.2) [31, 41]	38 (4) [32, 41]	0.151
Gestational age at birth (weeks)	39 (3) [31, 41]	39 (2.2) [31, 41]	38 (3) [34, 41]	0.186
Preterm birth	13 (21.3%)	6 (16.7%)	7 (28.0%)	0.456
Infant birthweight (g)	3064 (751) [1344, 4930]	3005 (799.8) [1344, 4930]	3130 (544) [2239, 4670]	0.592
Preeclampsia	9 (14.8%)	5 (13.9%)	4 (16.0%)	1.000
Gestational diabetes	10 (16.4%)	6 (16.7%)	4 (16.0%)	1.000
Diabetes	3 (4.92%)	1 (2.78%)	2 (8.00%)	0.562
Hypertension	2 (3.28%)	0 (0.00%)	2 (8.00%)	0.164
Gestational hypertension	7 (11.5%)	3 (8.33%)	4 (16.0%)	0.430

IQR, interquartile range; max, maximum; min, minimum.

The macroscopic and microscopic findings of placentas are summarized in Tables 2 and 3, respectively. Few statistically significant differences were found between case and control placentas. They were similar with respect to gross characteristics including disk weight (452 versus 432 g in negative and positive mothers, respectively), shape, and thickness. The median distance from the site of the cord insertion to the nearest disk margin was statistically significantly shorter among placentas from COVID-19 positive women (3.5 versus 6.0 cm, p = 0.006; Table 2). Placentas from COVID-19 positive mothers had a lower frequency of acute chorioamnionitis (16% versus 36.1%; Table 3), but this difference did not reach statistical significance (p = 0.152). Rates of chronic chorioamnionitis and villitis were similar between cases and controls. Placentas of COVID-19 positive mothers had a higher frequency of meconium (44.0% versus 25.0%; Table 3), but this difference was again not statistically significant (p = 0.201). Placentas from COVID-19 positive mothers were less likely to have intervillous thrombus in comparison to COVID-19 negative mothers (0% versus 19.4%; p = 0.035; Table 3). There is no significantly higher thrombotic or vascular events in placentas between the COVID-19 positive and negative mothers.

**Table 2:** Macroscopic (gross) placental characteristics from COVID-19 positive and negative mothers.

	Total N = 61	Negative N = 36	Positive N = 25	p-value
	N (%) or Median (IQR) [Min, Max]			
Placenta disk weight (g)	444 (180) [182, 895]	452.5 (187) [182, 862]	432 (175) [246, 895]	0.988
Length of disk (cm)	17 (4) [13.1, 27]	17.6 (5) [13.1, 22]	17 (4) [14, 27]	0.541
Width of disk (cm)	15 (3) [3, 20.5]	15 (2.9) [3, 20.5]	15 (2.3) [12, 20]	0.684
Thickness (cm)	2.5 (0.8) [2, 5]	2.5 (0.9) [2, 5]	2.5 (0.5) [2, 5]	0.818
Placental shape				0.802
Discoid	48 (78.7%)	29 (80.6%)	19 (76.0%)	
Ovoid	8 (13.1%)	5 (13.9%)	3 (12.0%)	
Irregular	5 (8.20%)	2 (5.56%)	3 (12.0%)	
Distance of cord from nearest margin (cm)	4.6 (3.5) [0.9, 9]	6 (3) [0.9, 9]	3.5 (2.5) [1, 9]	0.006
Number of vessels				1.000
2	1 (1.64%)	1 (2.78%)	0 (0.00%)	
3	60 (98.4%)	35 (97.2%)	25 (100%)	
Membrane insertion				0.093
Marginal	45 (75.0%)	24 (68.6%)	21 (84.0%)	
Peripheral	14 (23.3%)	11 (31.4%)	3 (12.0%)	
Circumvallate	1 (1.67%)	0 (0.00%)	1 (4.00%)	
Fetal surface fibrin present	34 (55.7%)	18 (50.0%)	16 (64.0%)	0.412
Maternal surface calcifications present	27 (44.3%)	17 (47.2%)	10 (40.0%)	0.767

IQR, interquartile range; max, maximum; min, minimum.

**Table 3:** Microscopic placental characteristics from COVID-19 positive and negative mothers.

	Total	Negative	Positive	p-value
	*N = 61*	*N = 36*	*N = 25*	
	N (%)				
Acute chorioamnionitis	17 (27.9%)	13 (36.1%)	4 (16.0%)	0.152
Acute funisitis	5 (8.2%)	4 (11.1%)	1 (4.0%)	0.640
Chronic chorioamnionitis	4 (6.56%)	3 (8.33%)	1 (4.00%)	0.638
Chronic villitis	3 (4.92%)	1 (2.78%)	2 (8.00%)	0.562
Meconium	20 (32.8%)	9 (25.0%)	11 (44.0%)	0.201
Maternal vascular malperfusion	6 (9.84%)	5 (13.9%)	1 (4.00%)	0.386
Abnormal fetal circulation	4 (6.56%)	3 (8.33%)	1 (4.00%)	0.638
Thrombosis and vascular changes				
Stem villous thrombosis				0.759
Absent	57 (93.4%)	33 (91.7%)	24 (96.0%)	
Acute	2 (3.28%)	2 (5.56%)	0 (0.00%)	
Chronic	2 (3.28%)	1 (2.78%)	1 (4.00%)	
Membranes maternal vessels:				0.759
Normal	57 (93.4%)	33 (91.7%)	24 (96.0%)	
Thrombosis	2 (3.28%)	1 (2.78%)	1 (4.00%)	
Mural muscle hypertrophy >50%	2 (3.28%)	2 (5.56%)	0 (0.00%)	
Intervillous thrombus	7 (11.5%)	7 (19.4%)	0 (0.00%)	0.035
Maternal vessels				0.508
Thrombosis	1 (1.64%)	1 (2.78%)	0 (0.00%)	
Mural muscle retention	2 (3.28%)	2 (5.56%)	0 (0.00%)	
**Miscellaneous**				
Membrane laminar necrosis	3 (4.92%)	2 (5.56%)	1 (4.00%)	1.000
Parenchyma: villi maturation				0.351
Appropriate	55 (90.2%)	32 (88.9%)	23 (92.0%)	
Immature	1 (1.64%)	0 (0.00%)	1 (4.00%)	
Hypermature	5 (8.20%)	4 (11.1%)	1 (4.00%)	
Villous edema	1 (1.64%)	0 (0.00%)	1 (4.00%)	0.410
Villous infarction	3 (4.92%)	3 (8.33%)	0 (0.00%)	0.262
Intervillous fibrinoid	6 (9.84%)	4 (11.1%)	2 (8.00%)	1.000

## Discussion

In this case-control study of 25 cases and 36 controls, we found rare indications that the placenta is impacted by maternal COVID-19 infection. However, we found that placenta from COVID-19 positive mothers had a statistically significantly shorter distance of cord insertion site to the disk margin, approaching marginal insertion in placentas from COVID-19 positive mothers. Similar findings have been reported in other studies [4,13], but the clinical significance of which remains debatable [33–35]. The tendency for a non-centrally inserted cord is unlikely to be related to maternal COVID-19 infection as the trajectory for cord insertion location is likely determined much earlier in pregnancy, prior to most of the women being diagnosed with COVID-19 in this study. Additionally, in this study there was a lower frequency of both disc lesions and intervillous thrombus among cases. It is likely that these differences are related to other clinical indications for placental pathologic review and not maternal COVID-19 infection.

This lack of significant differences in pathologic findings of cases and controls can be related to a number of possibilities; previous studies suggest that SARS-CoV-2 RNA and proteins are uncommonly found in placental tissue [4]. Another consideration includes the variability in host response to the virus resulting in variable disease severity, which was not collected in this study. In addition, the lack of pathologic findings in our study cohort may be the result of under-representation of otherwise uncommon pathology and larger cohorts and multiple studies are needed to further investigate the above reported findings. Furthermore, it is possible that COVID-19 infection during pregnancy only impacts placental characteristics when infection occurs at critical windows of exposure, such as early in gestation. Women in this study were tested at most 41 days prior to delivery, and the majority of mothers (84%) had a COVID-19 test on the day of delivery or up to 2 days prior, limiting the duration of exposure to SARS-CoV-2, and rendering chronic changes less likely. We cannot rule out the possibility of misclassification of the control women. Asymptomatic infection of the women in this study may have occurred earlier in pregnancy. Such differential misclassification would likely have biased results toward the null. However, both cases and controls were tested for COVID-19 using the same methodology reducing possible false negativity. Also, all patients delivered around the peak of the first wave of the COVID-19 pandemic in our geographic area, rendering infection early in pregnancy less likely.

SARS-CoV-2 continues to reveal itself as causing more of a systemic disease rather than being restricted to the pulmonary system. Its effect on placenta and its transplacental vertical transmission is yet to be fully understood. However, if the virus does indeed affect the placenta, it is not clear whether this is due to a direct effect of the virus on placental tissue or an effect induced indirectly through immunogenic mechanisms.

Direct effect of SARS-CoV-2 and its entry into the cell are mediated through the interaction of the viral structural spike (S) protein with the angiotensin-converting enzyme 2 (ACE2), a step promoted by the type 2 transmembrane serine protease (TMPRSS2), which cleaves ACE2 and activates SARS-CoV-2 S protein [2]. ACE2 and TMPRSS2 are expressed in a variety of host target cells, including alveolar epithelial cells. In placenta, ACE2 is expressed in invasive extravillous trophoblast and outer syncytiotrophoblast layer of placental villi, while there is only rare and weak TMPRSS2 expression [4,11,13]. This immunophenotype suggests that placentas can be infected with SARS-CoV-2 virus. Placental infection by SARS-CoV-2 has been investigated using immunohistochemistry for viral proteins, or in-situ hybridization or polymerase chain reaction for viral RNA. To date, the presence of the virus has been confirmed in 16 of 245 (6.5%) of tested placenta in the English literature [3–6,8–10,12–18,36–39] where these positive placentas show some association with the presence of chronic inflammation, e.g., intervillositis. Due to limited supply of testing supplies at our health system and prioritization of supplies for clinical care, we could not evaluate the placentas in this study for presence of virus.

The indirect systemic effect of SARS-CoV-2 on tissue, likely happening at a more advanced stage of the disease, is the result of multiple factors including the compromise of epithelial-endothelial barrier integrity, cytokine storm, activation of coagulations, and consumption of clotting factors [2]. In lung tissue, these manifest as chronic interstitial pneumonitis, edema, and acute respiratory distress syndrome, while systematically, they can result in thrombotic complications such as deep vein thrombosis, pulmonary embolism, thrombotic arterial complications (for example ischemic stroke and myocardial infarction), and end-organ failure [2]. Within the placenta, there are a few controlled studies that describe findings likely related to a systemic effect of the virus. Mulvey and colleagues reported a small series of 5 placentas from COVID-19 positive mothers showing evidence of fetal vascular malperfusion (fetal large vessel thrombosis within the chorionic plate and stem villi, intramural fibrin deposition, avascular villi, and villous stromal-vascular karyorrhexis) without unequivocal evidence of viral protein or RNA in the 5 placentas [7]. Shanes and colleagues studied the morphology of 15 placentas from mothers who tested positive for COVID-19 at variable intervals before delivery (0–2 days in 9, 6–7 days in 2, and 25–34 days in 4 cases) in comparison with findings from a historic control group of 17,479 placentas and a nested control group of 215 placentas with maternal history of melanoma [27]. In contrast to the previously discussed study, COVID-19 placentas from the second study showed no significant increase in fetal vascular malperfusion in comparison with all and nested historic control groups (12/15 COVID-19 placentas versus 9,596/17,479 all controls, p = 0.09 and 121/215 nested controls, p = 0.10). However, there was a significantly higher frequency of maternal vascular malperfusion in COVID-19 placentas in comparison to all and nested control groups (12/15 COVID-19 placentas versus 7,754/17,479 all controls, p = 0.046, and 59/215 nested controls, p = 0.001). These features included central (1/15) and peripheral (3/15) villous infarctions, villous agglutination (3/15), accelerated villous maturation (2/15), and decidual arteriopathy (7/15). Similarly, Smithgall and colleagues’ cohort exhibited significantly higher rates of features of maternal vascular malperfusion, namely villous agglutination and subchorionic thrombus in placentas from COVID-19 positive mothers than COVID-19 negative (21/51 versus 2/25, p = 0.003, and 9/51 versus 0/25, p = 0.026, respectively), but without significant inflammation or fetal vascular malperfusion. In other non-controlled studies and case reports, there are accounts of placentas from COVID-19 positive mothers with features of fetal vascular malperfusion [26] or maternal vascular malperfusion [37,40] and some with associated inflammation [5,10,12–14,36]. Finally, it remains a possibility that the findings reported here are due to chance as the sample size was small and a large number of histopathologic measures were evaluated. Additional studies are needed.

In summary, this study reports a cohort of placentas from mothers diagnosed with COVID-19 within the immediate prenatal period that showed few significant pathologic differences in comparison to placentas from COVID-19 negative mothers. We hypothesize that the reason behind the lack of significance is the rarity of pathologic effect of SARS-CoV-2 on placenta. However, this study does not examine long-term or early pregnancy impacts of maternal COVID-19 infection on placentas.
